# Supplementary material for: Disrupted brain connectivity in children treated with therapeutic hypothermia for neonatal encephalopathy
Source: Neuroimage Clin. 2021 Feb 10;30:102582. doi: 10.1016/j.nicl.2021.102582 (PMC7906894; doi:10.1016/j.nicl.2021.102582)
Supplement: Supplementary data 1 [file mmc1.pdf]

Supplementary Materials

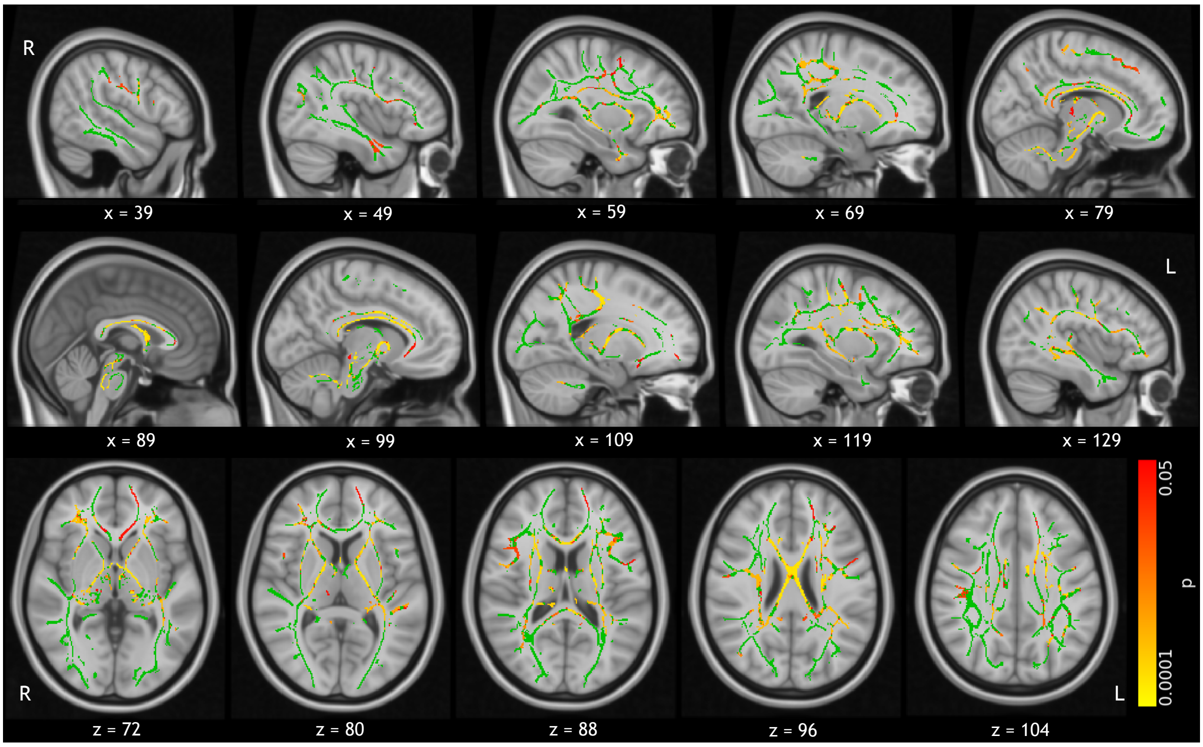

Supplementary Figure 1: TBSS results with covariates. FA on the white matter skeleton (green) was compared, with age, sex and deprivation index included as covariates in a general linear model. Significant results are indicated by the colour bar ($p < 0.05$, TFCE corrected). These are overlaid on the MNI standard template.

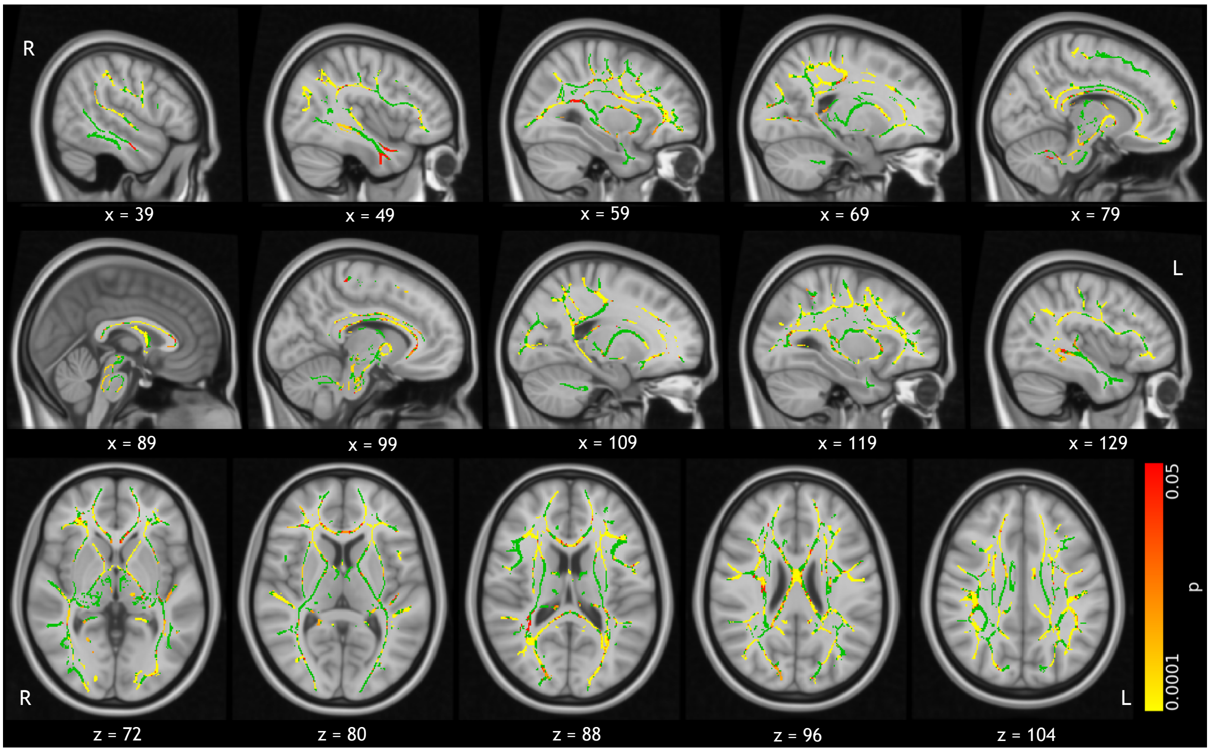

Supplementary Figure 2: Post hoc voxelwise correlation of FSIQ with FA in cases. TBSS was used to measure the correlation between FSIQ with FA on the white matter skeleton (green) was compared, with age and sex included as covariates in a general linear model. Significant results are indicated by the colour bar ($p < 0.05$, TFCE corrected). These are overlaid on the MNI standard template. Areas in which FA correlated with FSIQ include the corpus callosum, fornix, superior longitudinal fasciculus, anterior limbs of the internal capsule, and many other distributed areas of white matter. No significant correlations were found in controls.

Network Metric	Case	Control	Uncorrected p
Small-Worldness	0.8594	0.8506	0.2347
Average Node Strength	28.07	28.74	0.0769
Local Efficiency	0.3925	0.3993	0.1072
Global Efficiency	0.3885	0.3954	0.0927
Clustering Coefficient	0.3572	0.3652	0.0684
Modularity	0.0682	0.0655	0.3286
Characteristic Path Length	2.740	2.689	0.0829

Supplementary Table 1: Case-control comparison of network metrics. Network metrics were calculated from the FA-weighted structural network of each subject. All network metrics were found to be normally distributed within each group (Kolmogorov-Smirnov test; $p < 0.05$). Columns show average network metrics for each group and the p-value calculated by a two-tailed, unpaired t-test. None of these differences were significant before correction so Bonferroni corrected p-values are not shown.

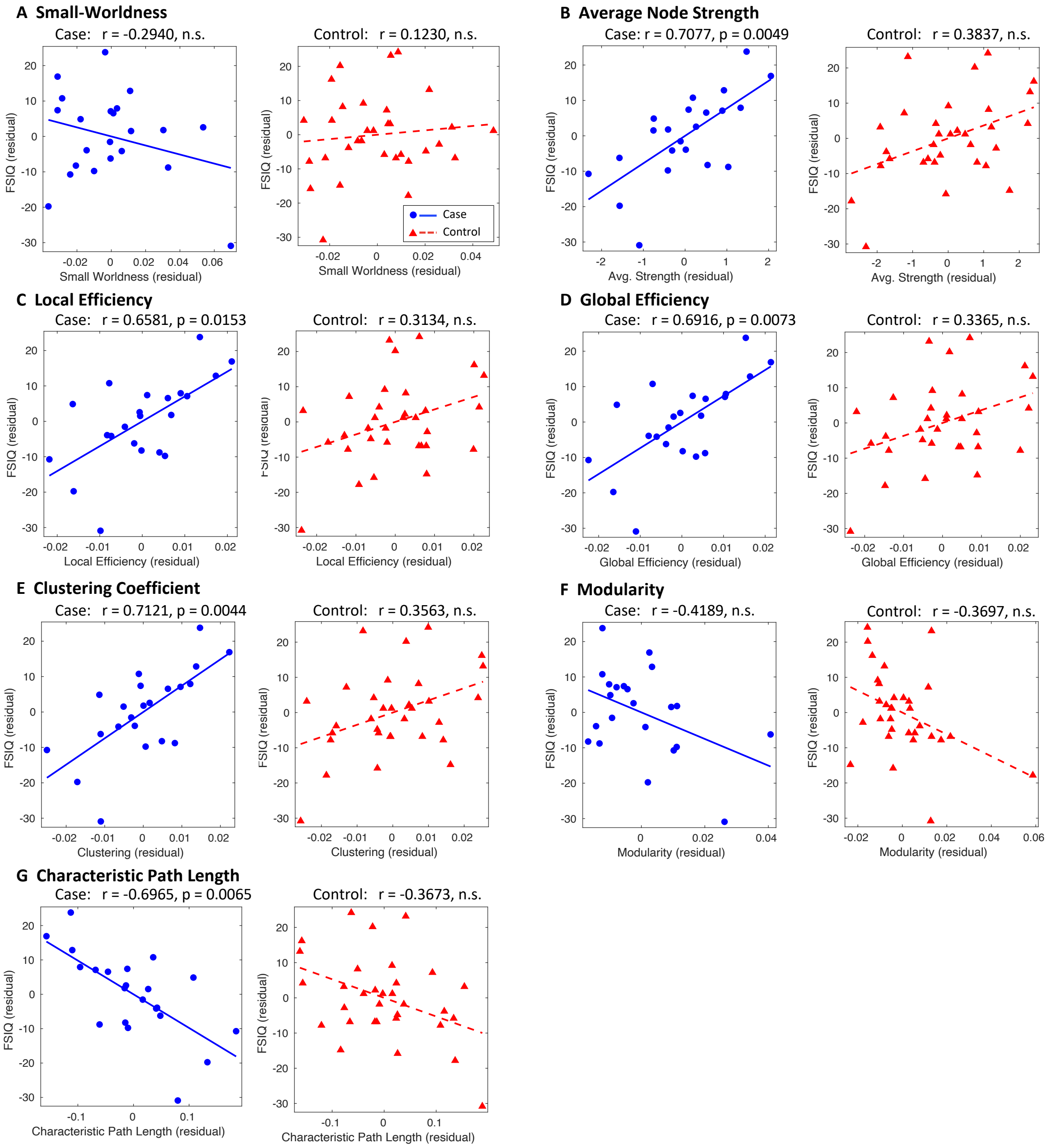

Supplementary Figure 3: Correlation of network metrics with FSIQ, independent of socio-economic status. Network metrics and FSIQ were controlled for age and sex and socio-economic status, as measured by the index of multiple deprivation (see Table 1, main text). Residuals are plotted for cases (blue circles) and controls (red triangles). These are fitted with a blue solid line and red dashed line for cases and controls respectively. Where $p > 0.05$, plots are labelled as not significant (n.s.). p-values are Bonferroni corrected for the number of correlations performed.

Case	Control	Age	Sex
Design			
1	0	6.75	0
0	1	7.25	1
…			
Contrast			
-1	1	0	0

Supplementary Table 2: Case-control comparison design matrix. The design matrix and contrast to test for reduced connectivity in cases compared to controls, with age and sex included as covariates, are shown.

Case	Control	Metric (case)	Metric (control)	Age	Sex
Design					
1	0	-7.5	0	6.75	0
0	1	0	5.47	7.25	1
…					
Contrasts					
0	0	1	-1	0	0
0	0	-1	1	0	0

Supplementary Table 3: Correlation design matrix. The design matrix and contrasts to test for stronger dependence of a given metric (e.g. FSIQ) on connectivity in cases than in controls, or a stronger dependence in controls than in cases, are shown. The metric is demeaned within each group and age and sex are included as covariates.

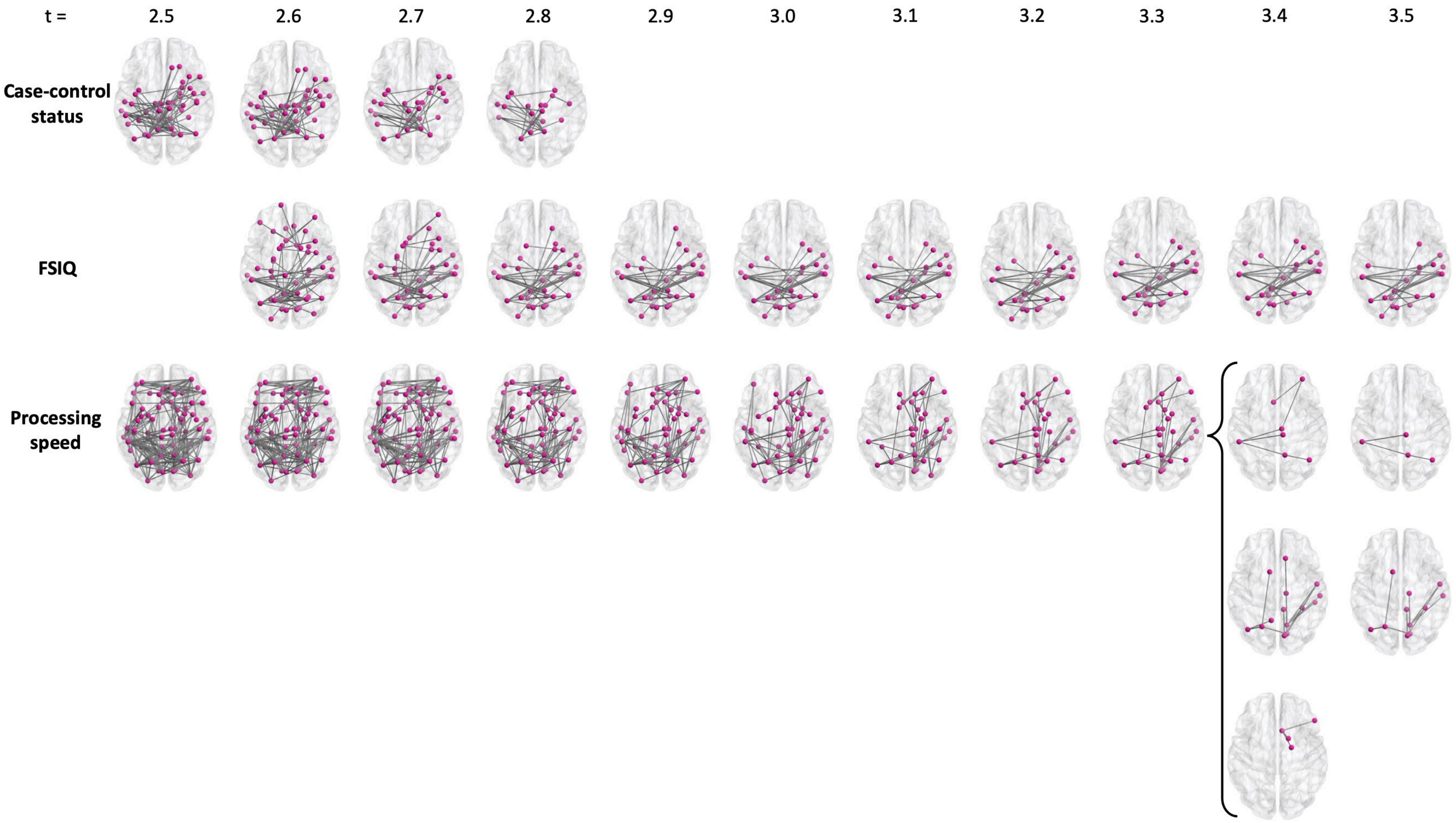

Supplementary Figure 4: Testing t-statistic thresholds for NBS. All networks shown are significant ($p < 0.05$). The primary thresholds chosen were: case-control status $t = 2.8$; FSIQ $t = 3.5$; processing speed $t = 3.3$.

Region	Connections	Function
R precuneus*	6	Visuo-spatial imagery, episodic memory retrieval and “self-processing” operations, namely first-person perspective taking and an experience of agency (Cavanna, 2007; Cavanna and Trimble, 2006).
L superior parietal gyrus	5	Attention and visuo-spatial perception.
L precuneus*	4	See R precuneus above.
L thalamus*	4	Relaying sensorimotor signals to the cortex.
L inferior temporal gyrus	3	Visual processing and visual object recognition.
L postcentral gyrus	2	Primary somatosensory cortex.
L precentral gyrus	2	Primary motor area.
R amygdala	2	Emotional behaviour.
R paracentral gyrus*	2	Sensorimotor functions of the lower limb.
L isthmus of the cingulate gyrus*	1	Connects the posterior cingulate cortex to the hippocampus and has a role in memory.
L paracentral gyrus*	1	See R paracentral gyrus above.
L superior temporal gyrus	1	Visual information integration (Karnath, 2001; Shen et al., 2017).
L insula	1	Sensorimotor as well as higher-level cognitive function (Uddin et al., 2017).
R thalamus*	1	See L thalamus above.
R putamen	1	Movement regulation.
R fusiform gyrus	1	Object and face recognition (Kleinhans et al., 2008; Pelphrey et al., 2007).
R isthmus of the cingulate gyrus*	1	See L isthmus above.
R posterior cingulate gyrus	1	Internally directed thought (Leech et al., 2011) and task management (Pearson et al., 2011).
R transverse temporal gyrus	1	Part of the auditory cortex.

Supplementary Table 4: Nodes in the case-control status subnetwork. These are listed by the number of connections they make within this subnetwork, and associated function. * nodes which are present bilaterally in the subnetwork.

Region	Connections	Function
L precuneus cortex	4	Visuo-spatial imagery and episodic memory (see Supplementary Table 4).
L superior parietal gyrus*	4	Attention and visuo-spatial perception.
L supramarginal gyrus*	4	Visual word recognition and semantic word processing (Stoeckel et al., 2009).
R supramarginal gyrus*	4	See L supramarginal gyrus above.
R parahippocampal gyrus	4	Memory encoding and retrieval (Eichenbaum, 2000).
L inferior parietal gyrus*	3	Association area at the temporo-parietal junction (Igelström and Graziano, 2017).
R superior parietal gyrus*	2	See L superior parietal gyrus above.
R inferior parietal gyrus*	2	See L inferior parietal gyrus above.
L inferior temporal gyrus*	2	Visual processing and visual object recognition.
R superior temporal gyrus	2	Visual information integration (Karnath, 2001; Shen et al., 2017).
R inferior temporal gyrus*	1	See R inferior temporal gyrus above.
L cuneus cortex	1	Basic visual processing.
L lateral occipital gyrus	1	Visual object recognition (Grill-Spector et al., 1999).
L lingual gyrus	1	Visual association cortex.
L pericalcarine cortex*	1	Visual association cortex.
R pericalcarine cortex*	1	See L pericalcarine cortex above.
R hippocampus	1	Memory formation and spatial navigation (Eichenbaum, 2000).
R banks of superior temporal sulcus	1	Visual attention and goal-direction action (Shultz et al., 2011).
R isthmus of the cingulate gyrus	1	Connects the posterior cingulate cortex to the hippocampus and has a role in memory.
R middle temporal gyrus	1	Semantic memory processing.
R postcentral gyrus*	1	Primary somatosensory cortex.
L postcentral gyrus*	1	See R postcentral gyrus above.
R temporal pole	1	Social and emotional processing.

Supplementary Table 5: Nodes in the FSIQ subnetwork. These are listed by the number of connections they make within this subnetwork, and associated function. * nodes which are present bilaterally in the subnetwork.

Region	Connections	Function
R lingual gyrus	9	Visual association cortex.
L superior parietal gyrus*	4	Attention and visuo-spatial perception.
R cuneus cortex	4	Basic visual processing.
L supramarginal gyrus	3	Visual word recognition and semantic word processing (Stoeckel et al., 2009).
R caudal anterior cingulate gyrus*	3	Cognitive processes such as attention, salience and interference (Bush et al., 2000).
R pericalcarine cortex	3	Visual association cortex.
R precuneus*	3	Visuo-spatial imagery and episodic memory (see Supplementary Table 4).
R rostral middle frontal gyrus	3	Executive function; the region, as defined by Freesurfer, comprises the dorsolateral prefrontal cortex (Desikan et al., 2006; Kikinis et al., 2010), which is involved in working memory (Barbey et al., 2013) and attention and attention (Japee et al., 2015; Rosen et al., 1999).
R superior temporal gyrus	3	Visual information integration (Karnath, 2001; Shen et al., 2017).
L inferior parietal gyrus*	2	Association area at the temporo-parietal junction (Igelström and Graziano, 2017).
L accumbens area	2	Motor function and reward.
R thalamus	2	Relaying sensorimotor signals to the cortex.
R pallidum	2	Motor function and reward.
R posterior cingulate gyrus	2	Internally directed thought (Leech et al., 2011) and task management (Pearson et al., 2011).
R superior parietal gyrus*	2	See L superior parietal gyrus above.
L caudal anterior cingulate gyrus*	1	See R caudal anterior cingulate gyrus above.
L precuneus*	1	See R precuneus above.
R caudate	1	Goal-directed action.
R putamen	1	Movement regulation.
R hippocampus	1	Memory formation and spatial navigation (Eichenbaum, 2000).
R fusiform gyrus	1	Object and face recognition (Kleinhans et al., 2008; Pelphrey et al., 2007).
R inferior parietal gyrus*	1	See L inferior parietal gyrus above.
R inferior temporal gyrus	1	Visual processing and visual object recognition.
R isthmus of the cingulate gyrus	1	Connects the posterior cingulate cortex to the hippocampus and has a role in memory.
R middle temporal gyrus	1	Semantic memory processing.
R paracentral gyrus	1	Sensorimotor functions of the lower limb.
R pars triangularis	1	Semantic processing.
R superior frontal gyrus	1	Working memory and attention (Li et al., 2013), and contains the supplementary motor area.

Supplementary Table 6: Nodes in the processing speed subnetwork. These are listed by the number of connections they make within this subnetwork, and associated function. * nodes which are present bilaterally in the subnetwork.

Frontal		***Limbic***	
CMFG	Caudal middle frontal gyrus	AC	Accumbens area
FP	Frontal pole	AM	Amygdala
LOFG	Lateral orbital frontal gyrus	CACG	Caudal anterior cingulate gyrus
MOFG	Medial orbital frontal gyrus	HI	Hippocampus
PaCG	Paracentral gyrus	ICG	Isthmus of the cingulate gyrus
POP	Pars opercularis	IN	Insula
POR	Pars orbitalis	PCG	Posterior cingulate gyrus
PTR	Pars triangularis	PHIG	Parahippocampal gyrus
PrCG	Precentral gyrus	RACG	Rostral anterior cingulate gyrus
RMFG	Rostral middle frontal gyrus		
SFG	Superior frontal gyrus		
Parietal		***Occipital***	
IPG	Inferior temporal gyrus	CU	Cuneus cortex
PCU	Precuneus cortex	LG	Lingual gyrus
PoCG	Postcentral gyrus	LOG	Lateral occipital gyrus
SMG	Supramarginal gyrus	PCAL	Pericalcarine cortex
SPG	Superior parietal gyrus		
Temporal		***Subcortical***	
BSTS	Banks of the superior temporal sulcus	PA	Pallidum
EC	Entorhinal cortex	PU	Putamen
FG	Fusiform gyrus	TH	Thalamus
ITG	Inferior temporal gyrus		
MTG	Middle temporal gyrus	CER	Cerebellum
STG	Superior temporal gyrus		
TP	Temporal pole		
TTG	Tranverse temporal gyrus		

Supplementary Table 7: Node label abbreviations.

References

Barbey AK, Koenigs M, Grafman J. 2013. Dorsolateral prefrontal contributions to human working memory. *Cortex* **49**:1195–1205. doi:10.1016/j.cortex.2012.05.022

Bush G, Luu P, Posner MI. 2000. Cognitive and emotional influences in anterior cingulate cortex. *Trends Cogn Sci* **4**:215–222. doi:10.1016/S1364-6613(00)01483-2

Cavanna AE. 2007. The Precuneus and Consciousness. *CNS Spectr* **12**:545–552. doi:10.1017/S1092852900021295

Cavanna AE, Trimble MR. 2006. The precuneus: a review of its functional anatomy and behavioural correlates. *Brain* **129**:564–583. doi:10.1093/brain/awl004

Desikan RS, Ségonne F, Fischl B, Quinn BT, Dickerson BC, Blacker D, Buckner RL, Dale AM, Maguire RP, Hyman BT, Albert MS, Killiany RJ. 2006. An automated labeling

system for subdividing the human cerebral cortex on MRI scans into gyral based regions of interest. *Neuroimage* **31**:968–980. doi:10.1016/j.neuroimage.2006.01.021

Eichenbaum H. 2000. A cortical–hippocampal system for declarative memory. *Nat Rev Neurosci* **1**:41–50. doi:10.1038/35036213

Grill-Spector K, Kushnir T, Edelman S, Avidan G, Itzchak Y, Malach R. 1999. Differential Processing of Objects under Various Viewing Conditions in the Human Lateral Occipital Complex. *Neuron* **24**:187–203. doi:10.1016/S0896-6273(00)80832-6

Igelström KM, Graziano MSA. 2017. The inferior parietal lobule and temporoparietal junction: A network perspective. *Neuropsychologia* **105**:70–83. doi:10.1016/j.neuropsychologia.2017.01.001

Japee S, Holiday K, Satyshur MD, Mukai I, Ungerleider LG. 2015. A role of right middle frontal gyrus in reorienting of attention: a case study. *Front Syst Neurosci* **9**. doi:10.3389/fnsys.2015.00023

Karnath H-O. 2001. New insights into the functions of the superior temporal cortex. *Nat Rev Neurosci* **2**:568–576. doi:10.1038/35086057

Kikinis Z, Fallon JH, Niznikiewicz M, Nestor P, Davidson C, Bobrow L, Pelavin PE, Fischl B, Yendiki A, McCarley RW, Kikinis R, Kubicki M, Shenton ME. 2010. Gray matter volume reduction in rostral middle frontal gyrus in patients with chronic schizophrenia. *Schizophr Res* **123**:153–159. doi:10.1016/j.schres.2010.07.027

Kleinhans NM, Richards T, Sterling L, Stegbauer KC, Mahurin R, Johnson LC, Greenson J, Dawson G, Aylward E. 2008. Abnormal functional connectivity in autism spectrum disorders during face processing. *Brain* **131**:1000–1012. doi:10.1093/brain/awm334

Leech R, Kamourieh S, Beckmann CF, Sharp DJ. 2011. Fractionating the Default Mode Network: Distinct Contributions of the Ventral and Dorsal Posterior Cingulate Cortex to Cognitive Control. *J Neurosci* **31**:3217–3224. doi:10.1523/JNEUROSCI.5626-10.2011

Li W, Qin W, Liu H, Fan L, Wang J, Jiang T, Yu C. 2013. Subregions of the human superior frontal gyrus and their connections. *Neuroimage* **78**:46–58. doi:10.1016/j.neuroimage.2013.04.011

Pearson JM, Heilbronner SR, Barack DL, Hayden BY, Platt ML. 2011. Posterior cingulate

cortex: adapting behavior to a changing world. *Trends Cogn Sci* **15**:143–151. doi:10.1016/j.tics.2011.02.002

Pelphrey KA, Morris JP, McCarthy G, LaBar KS. 2007. Perception of dynamic changes in facial affect and identity in autism. *Soc Cogn Affect Neurosci* **2**:140–149. doi:10.1093/scan/nsm010

Rosen AC, Rao SM, Caffarra P, Scaglioni A, Bobholz JA, Woodley SJ, Hammeke TA, Cunningham JM, Prieto TE, Binder JR. 1999. Neural Basis of Endogenous and Exogenous Spatial Orienting: A Functional MRI Study. *J Cogn Neurosci* **11**:135–152. doi:10.1162/089892999563283

Shen W, Yuan Y, Liu C, Luo J. 2017. The roles of the temporal lobe in creative insight: an integrated review. *Think Reason* **23**:321–375. doi:10.1080/13546783.2017.1308885

Shultz S, Lee SM, Pelphrey K, McCarthy G. 2011. The posterior superior temporal sulcus is sensitive to the outcome of human and non-human goal-directed actions. *Soc Cogn Affect Neurosci* **6**:602–611. doi:10.1093/scan/nsq087

Stoeckel C, Gough PM, Watkins KE, Devlin JT. 2009. Supramarginal gyrus involvement in visual word recognition. *Cortex* **45**:1091–1096. doi:10.1016/j.cortex.2008.12.004

Uddin LQ, Nomi JS, Hébert-Seropian B, Ghaziri J, Boucher O. 2017. Structure and Function of the Human Insula. *J Clin Neurophysiol* **34**:300–306. doi:10.1097/WNP.0000000000000377

Zalesky A, Cocchi L, Fornito A, Murray MM, Bullmore E. 2012. Connectivity differences in brain networks. *Neuroimage* **60**:1055–1062. doi:10.1016/j.neuroimage.2012.01.068

Zalesky A, Fornito A, Harding IH, Cocchi L, Yücel M, Pantelis C, Bullmore ET. 2010. Whole-brain anatomical networks: Does the choice of nodes matter? *Neuroimage* **50**:970–983. doi:10.1016/j.neuroimage.2009.12.027
